# Postpartum depression in Vietnam: a scoping review of symptoms, consequences, and management

**DOI:** 10.1186/s12905-023-02519-5

**Published:** 2023-07-26

**Authors:** Huyen Thi Hoa Nguyen, Phuong Anh Hoang, Thi Kim Ly Do, Andrew W. Taylor-Robinson, Thi Thanh Huong Nguyen

**Affiliations:** 1 College of Health Sciences, VinUniversity, Vinhomes Ocean Park, Hanoi, Da Ton, Gia Lam 100000 Vietnam; 2 Faculty of Health, University of Technology Sydney, 15 Broadway, Ultimo, NSW 2007 Australia; 3 Faculty of Nursing and Midwifery, Hanoi Medical University, No 1 Ton That Tung, Dong Da, 100000 Hanoi, Vietnam; 4 University Medical Center Schleswig-Holstein, Lübeck campus, Ratzeburger Allee 96, Lübeck, 23562 Germany

**Keywords:** Postpartum depression, Symptom, Consequence, Management, Vietnam

## Abstract

**Background:**

Postpartum depression (PPD) is a major health issue that can affect both mothers and their newborn children. In Vietnam, approximately 20% of mothers suffer from PPD. However, there is a lack of synthesized evidence regarding the case management of PPD in the Vietnamese context. A review of early symptoms, consequences, and management strategies of PPD will help to inform best practices to reduce complications and shorten the recovery time after parturition.

**Methods:**

This scoping review aims to analyze and synthesize the findings of studies on PPD examining the symptoms, consequences, and management strategies among Vietnamese women. MEDLINE, CINAHL, PubMed, ScienceDirect, EBSCOHost, Google Scholar, and a networked digital library of projects, theses, and dissertations published between 2010 and 2022 in Vietnam were accessed following search terms including “Vietnam”, “depression”, “postpartum”, “symptom/experience”, “consequence”, and “management”.

**Findings:**

The most-reported symptoms were sadness, tiredness, the feeling of being ignored, lack of interest in the baby, reduced appetite, and sleep disturbance. The recognized consequences were child stunting and slow growth, without mentioning its long-term effects on mothers. Our findings indicated that PPD in Vietnam has not been sufficiently managed; mothers tend to seek help from ‘fortune-tellers’ or ‘word-of-mouth’ practices rather than from evidence-based modern medicine.

**Conclusion:**

This scoping review provides an initial stage of PPD symptoms, consequences, and management along with facilitating an interventional program to support this vulnerable group of women. A large survey of Vietnamese mothers' symptoms, effects, and management strategies is needed.

## Statement of Significance (SOS)

### Problem or issue

Although postpartum depression (PPD) has long been recognized as a maternal health problem resulting in burdens for both mother and infant, there is still a lack of evidence regarding symptoms, consequences, and how it is managed in the Vietnamese setting.

### What is already known?

Studies examining PPD in Vietnam have revealed a high prevalence, approximately 20%, of women suffering from PPD. Common symptoms of PPD were also reported previously, but not a scoping review, in Vietnamese or English, of the early recognition of symptoms, consequences, and effective management strategies.

### What this paper adds

This study is the first scoping review synthesizing common symptoms, their consequences, and available management strategies for women in Vietnam. The findings indicate the importance of early detection of PPD symptoms in nursing practice. The paper offers a comprehensive evaluation of evidence in the Vietnamese context that supports the development of health education programs for the community and establishes a framework for the management of PPD.

## Background

Postpartum depression (PPD), also defined as non-psychotic depression, with onset within 4–6 weeks of giving birth up to six months or even later, is a major maternal health problem [1–3]. The prevalence of PPD worldwide is around 15% [3–5], while in Asian countries the reported range is from 3.5% to 63.3% [6]. In Vietnam, the PPD rate is approximately 20% [7–10]. Untreated PPD affects not only a mother’s mental and physical health but also their child’s development [11] and family relationships, particularly mother–child bonding [4]. As such, management of PPD is essential to improving the health outcomes of both mother and child [12, 13].

Most of the recent research on PPD has focused heavily on the prevalence and risk factors of PPD [14, 15] in order to improve community awareness regarding the importance of preventing PPD. The fact that depression is often overlooked during pregnancy or the postpartum period emphasizes the need for timely screening of obstetrical and primary care symptoms [16]. More importantly, the diagnostic guidelines of PPD [2] state that some symptoms should be linked with moderate to severe intensity and distress. This generated questions for nurses about how to recognize early and manage the common symptoms of pregnant women diagnosed with PPD [17]. Despite the fact that symptoms of PPD are consistent across nations, early screening, which is influenced by the sociopolitical perceptions of mental health illness and evidence-based information [18, 19], would reduce the risk of PPD’s potentially negative effects on both the mother and the infant. In Vietnam, the National Guidelines on Reproductive Healthcare services in 2016 clearly outlined the caring instructions for healthcare workers in 1-day, 1-week and 6-week after birth but none of the emphasis on mental screening was mentioned [20], which limited the nursing practices on mental care and early screening for postpartum women. Additionally, inadequate acknowledgment and community understanding of mental health illnesses, including PPD, as well as societal stigmatization of those who suffer from them, discourage women, family members, and the community from reporting and recognizing symptoms in a timely and proper manner [21, 22]. Moreover, mental diseases are frequently disregarded and, when they do, are blamed on fate or faults the person did in a past life. Symptoms, consequences, and follow-up of PPD have also not yet been systematically summarized to provide a comprehensive view of PPD management in the context of Vietnam. Such information could be used in combination with evaluating the importance of early screening for PPD and developing an appropriate intervention strategy to target women suffering from PPD. This knowledge gap highlights an urgent need to conduct a scoping review to synthesize the current findings on symptoms and consequences of PPD, especially among Vietnamese women to understand the current state and recommend the most suitable intervention programs.

The most recent review of PPD was published in 2012, which synthesized articles from different countries regarding the specific signs and symptoms, appropriate screening methods, and proper treatment [23]. However, this covered major traits of PPD across the world instead of the Vietnamese context with considerations of its socioeconomic differences. Additionally, changes and updates in medical treatment and symptoms management during the previous decade require a timely reappraisal of evidence. This scoping review of PPD in Vietnam, therefore, provides an updated overview of this important maternal health condition. The specific aim was to analyze the findings of studies examining symptoms, consequences, and management strategies to understand the effects of PPD and how it is managed in women who were Vietnamese nationals and living in Vietnam.

## Methods

### Search strategies

**Fig. 1:**
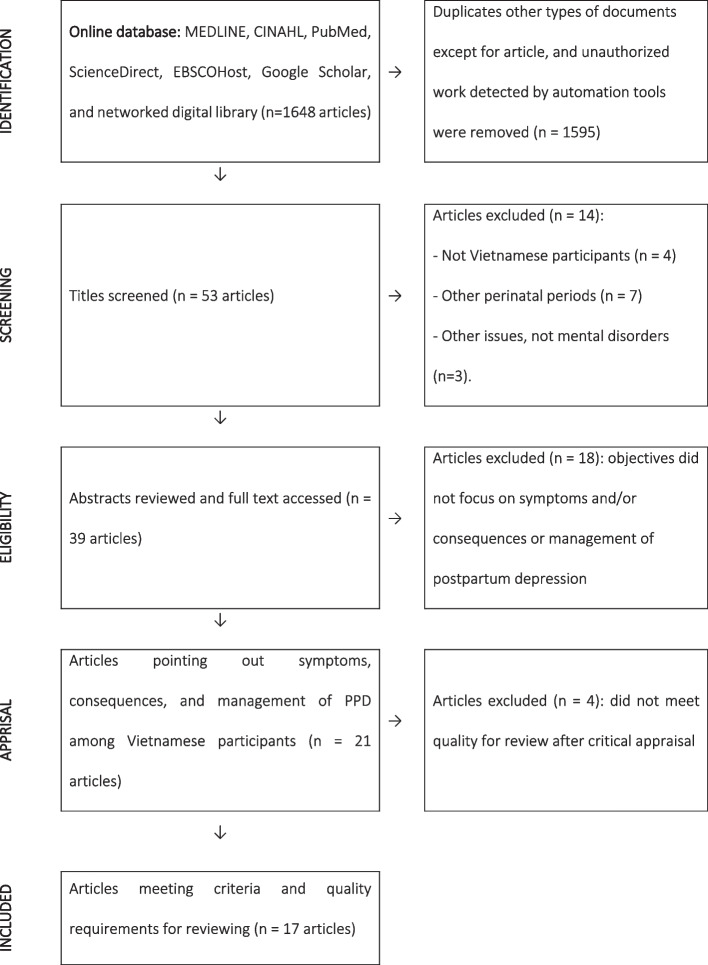
Search process

A literature search was conducted manually to review reported studies among Vietnamese women using the following online databases: MEDLINE, CINAHL, PubMed, ScienceDirect, EBSCOHost, Google Scholar, and a networked digital library of projects, theses, and dissertations in Vietnam. Search terms in both Vietnamese and English, including “Vietnam”, “depression”, “postpartum”, “symptom/experience”, “consequence”, and “management”, were used separately and as combinations during the search. All published and unpublished projects matching these key terms were included for screening. Each published article cited other studies that were also checked for relevance. Following the search, the title and abstract of every study were filtered and reviewed before being included for critical appraisal. The review process is presented in Fig. 1.

### Inclusion and exclusion criteria

Articles were included if the reported study fulfilled each of the following criteria: (i) examined symptoms, experiences, consequences, or management strategies of PPD; (ii) was published between 2010 and 2022; and (iii) was conducted among Vietnamese participants regardless of quantitative or qualitative method. Findings from both quantitative and qualitative perspectives can provide a much broader summary of the literature to accomplish the aims.

### Analysis process

Eligible articles were reviewed before the final decision of inclusion or exclusion. All titles, abstracts, and full texts were examined to ensure relevance to the research topic. Suitable papers were retained in the review list, while irrelevant ones were removed. This step was followed by a detailed process of reading and analyzing the full text of qualified publications that was performed by two researchers independently of each other to ensure impartiality and preciseness. The quality of these articles was assessed using the critical appraisal checklists of the JBI (Joanna Briggs Institution). At the completion of the analysis, the content of the finally included publications was categorized into a literature matrix. Table 1 shows the literature review matrix of 17 articles included in this scoping review.

**Table 1:** Literature matrix of 17 included articles

No	Title	Year – Author	Objectives	Participants	Design	Sample size	Setting	Tools	Postnatal time point screened	Symptoms	Consequence/ Management
1	Prevalence, nature, severity and correlates of postpartum depressive symptoms in Vietnam	Fisher, J. R. W., M. M. Morrow, N. T. Nhu Ngoc, and L. T. Hoang Anh (2004) [24]	To examine depressive symptomatology in women after childbirth in Ho Chi Minh City, Vietnam	Mothers of infants aged ± six weeks attending well-baby clinics	A cross-sectional quantitative study	506	Hung Vuong Obstetrics and Gynaecology Hospital and the Maternal, Child Health and Family Planning Center of Ho Chi Minh City	EPDS	6 weeks	Difficulty swallowing Heavy heart Breathing difficulty Heart palpitations Body feels cold Nausea Headaches Flatulence Constipation/diarrhea General worrying Nighttime waking apart from infant care Difficulty falling asleep Severe fatigue	
2	Domestic Moods: Maternal Mental Health in Northern Vietnam	Gammeltoft, Tine M. (2018) [25]	To develop the concept of domestic mood as an important concept for mental health research	Pregnant women	Part of a cohort study	30	Antenatal care facilities, Đông Anh district, Hanoi	EPDS. Cutting point/scale 30 points (10 items)	4 times: at enrolment (at a gestational age of less than 24 weeks); at a gestational age of 30–34 weeks; 24–48 h after delivery; and 4–12 weeks after delivery	Living under pressure The psychic force of household tensions The weight of kinship conflicts	
3	Emotional violence and maternal mental health: a qualitative study among women in northern Vietnam	Trần Thơ Nhị, Nguyễn Thị Thúy Hạnh & Tine M. Gammeltoft (2018) [26]	To explore Vietnamese women’s experiences of emotional partner violence and their perceptions of the implications of such violence for their mental health	10 pregnant women and 10 recently postpartum mothers	Qualitative study	20	Dong Anh District, Hanoi	EPDS	Either during pregnancy or after birth	Key dimensions of emotional partner violence: being ignored; being denied support; and exposure to controlling behaviors. These experiences affected the women’s sense of well-being profoundly, causing sadness and distress	
4	Postpartum Depressive Symptoms and Associated Factors in Married Women: A Cross-sectional Study in Danang City, Vietnam	Van Vo, Thang, Thi Kim Duong Hoa, and Tuyen Dinh Hoang. (2017) [8]	To (1) estimate the prevalence of PPD symptoms among married women in one Vietnam city (Danang), and (2) identify social and personal factors associated with postpartum depressive symptoms	Women who gave birth 4 weeks to 6 months prior to being interviewed	Cross-sectional study	600	Hai Chau District, Danang, Vietnam	EPDS (cutoff point of 12/13)	4-week to 6-months postnatal	Among women with postpartum depression symptoms, 37.9% had suicidal thoughts in the previous seven days (95% CI: 28.96–46.89)	
5	Symptom Endorsement and Sociodemographic Correlates of Postnatal Distress in Three Low Income Countries	Nguyen, Amanda J., Emily E. Haroz, Tamar Mendelson, and Judith Bass. (2016) [9]	To 1) compare endorsement of specific symptoms by mothers meeting criteria for maternal distress in these three settings, and (2) evaluate the consistency of associations between maternal distress and recognized risk factors	5647 mothers in Ethiopia, India (Andhra Pradesh), and Vietnam participating in an ongoing cohort study (Young Lives)	Cross-sectional, secondary analysis	1855 Vietnamese mothers among 5647 participants	Ethiopia, India, and Vietnam	SRQ. Probable clinically relevant maternal distress was classified by the YL team using the Self-Reporting Questionnaire-20 Items (SRQ-20), consists of 20 yes/no questions (no = 0; yes = 1), score ranging from 0 to 20 with higher scores indicating greater severity. cutoff of 8 or higher	6–18 months postnatal	Feeling nervous, being easily tired, headaches, being tired all the time, being unhappy, and poor appetite, feeling worthless, feeling unable to go on, and being unable to play a useful part in life	
6	Feelings of women who first-time deliver	Tran Thi Phuong Thao and Truong Thi Khanh Ha (2010) [27]	To explore the emotions of first-time mothers and factors that influence mothers’ emotions to make recommendations to help mothers and family members better understand the complex emotions of mothers in certain childcare and parenting situations	Mothers (20 to 38 years old) with children between 1.5 and 2 years old	A mix of qualitative and quantitative study, questionnaire survey, opinion poll, in-depth interview	Interview: 10 mothers; survey: 60 mothers	Hoan Kiem, Thanh Tri, and Thanh Xuan Districts, Hanoi	In-depth interview using open-ended questions. Percentage survey using self-designed questionnaire consisting of 24 questions, Likert scale from 1 to 4 (one question) and from 1 to 5 (some questions)	Over 1 year and 2 years postnatal	During the period from 3 to 9 months, when the baby's crying often makes the mothers "sad and worried" (20%); “sad and tired, never know when to let go of this phase” (43.3%). Qal: stuck, bored with raising children, frustrated with her husband and everyone, unable to hold back tears When the husband does not know how to share: 13.3% of them "feel very sad". Qal: often angry, emotional, or thinking about it, even having a night of thinking and not being able to sleep, feeling sorry for being alone with the child, my husband rarely asks about mother and daughter Sick child: 30% of young mothers feel "stressed out"	
7	Postnatal depressive symptoms display marked similarities across continents	Wesselhoeft, Rikke, Frederikke Kjerulff Madsen, Mia Beck Lichtenstein, Christian Sibbersen, Rachel Manongi, Declare L. Mushi, Hanh Thi Thuy Nguyen, et al. (2020) [28]	To (1) examine and compare the factor structure of postnatal depressive symptoms measured by EPDS in postpartum women from Denmark, Vietnam and Tanzania; (2) test the fit of the EPDS 3-factor structure identified in a study by Chiu et al.; (3) to examine if country of origin or education level predict high total EPDS score and (4) investigate whether there are differences in expression (frequency and severity) of specific depressive symptoms based on country of origin or education level	Women who were part of one of the three pregnancy cohorts: Denmark- early pregnancy and up until 2.5 months postpartum; Vietnam and Tanzania: early pregnancy and up until gestational age 24 weeks in	A cross-sectional study	4516 (Vietnam: 1,278)	Denmark, Vietnam, and Tanzania	EPDS cut off point of 12 and above	40–90 days postpartum	High proportions for symptom in Vietnamese population: Self-blame (28.8%); worry (27.3%) scared (10.3%) overwhelmed (16.4%) difficult to sleep (12.2%)	The highest level of education (level 3) was associated with a significantly lower EPDS total score, when adjusting for country (*p* < 0.001). We also tested the association between education duration (length in years) and EPDS total score, which confirmed a negative correlation (coefficient = 0.015, SE = 0.005, *p* = 0.0025). EPDS score was associated with stunting (low length for age) (mean EPDS score 9.3 stunting, 7.6 no stunting) (t – 2.23, *p* = 0.03)
8	Associations of Psychosocial Factors with Maternal Confidence Among Japanese and Vietnamese Mothers	Goto, Aya, Quang Vinh Nguyen, Thi Tu Van Nguyen, Nghiem Minh Pham, Thi Mong Thuy Chung, Huu Phuc Trinh, Junko Yabe, Hitomi Sasaki, and Seiji Yasumura. (2010) [7]	To investigate the prevalence and associated sociodemographic, parenting, and psychological characteristics of low maternal confidence in child rearing among them	Mother that had children between 1-month and 3-month-old	Cross-sectional study	294 Vietnamese women	Tu Du Obstetrical and Gynecological Hospital, where?	GSE scale and a two-question case-finding instrument (Whooley 1997)	1–3 month postnatal	Feeling I am abusing my child (10%); don’t have time to interact with child in relaxed mood (9%)	
9	Postpartum change in common mental disorders among rural Vietnamese women: incidence, recovery and risk and protective factors	Nguyen, Trang Thu, Thach Duc Tran, Tuan Tran, Buoi La, Hau Nguyen, and Jane Fisher (2015) [29]	To determine the incidence and rates of recovery from common mental disorders (CMD) among rural Vietnamese women and the risk and protective factors associated with these outcomes from the perinatal period to 15 months after giving birth	Mothers in the last 3 months of pregnancy or the first 4–6 weeks postpartum; follow up to 15 months later	A population-based prospective study	211	Rural and urban areas of Vietnam, including Ha Nam province	DSM-IV. assessed by psychiatrist administered Structured Clinical Interview for Disorders	1 year postnatal		More than two-thirds of women with CMD at baseline had recovered by the follow-up assessment. The probability (incidence) of a woman who was healthy at baseline having symptoms meeting diagnostic criteria for a CMD at follow-up (0.13, 95% CI 0.08–0.19) was significantly lower than the probability of a woman having a CMD at baseline and experiencing at least one CMD at follow-up (0.30, 95% CI 0.20–0.40). Overall, 70% (95% CI 59–80) of women who experienced a perinatal CMD recovered in the following year None received formal mental healthcare
10	The social contexts of depression during motherhood: A study of explanatory models in Vietnam	Niemi, Maria E., Torkel Falkenberg, Mai T. T. Nguyen, Minh T. N. Nguyen, Vikram Patel, and Elisabeth Faxelid. (2010) [30]	To elicit Illness Explanatory Models (EMs) of depression and postnatal depression from nine mothers and nine health workers	Nine mothers and nine health workers who meet mothers during the pregnancy/postpartum period	Qualitative	9 mothers and 9 health workers	A community health center in Ba Vi, a district in Ha Tay province of northern Vietnam	DSM. The semi-structured interview schedule was designed in accordance with the four main categories that consist of Kleinman's illness explanatory model framework (Kleinman, 1980)	Mothers with older children (adults or older than 1 year old)	In Vietnam, Confucianism, Buddhism and Taoism have carried major impacts in creating a holistic thinking where clear distinctions between physical and psychological symptoms are not made (Phan and Silvoe, 1999). Phan and Silvoe (1999) have shown that the Cartesian mind/body dualistic fashion of thinking that underlies Western psychiatric nosology does thus not necessarily coincide with this holistic view. They think that they are useless and don't want to become a burden for others	Advice: seeking formal help, advice against medical help, others' involvement in help and self-help. Psychiatric treatment and care were seldom recommended. (Formal help: somatic medical help was most not advised to treat the depression itself, but to treat a physical illness or disease that was considered the underlying cause for the depression; Psychiatric treatment was the second most common advice given by over half of the health workers. Advice to seek formal help for depression itself, and not its causes, was rarely expressed by the mothers. However, seeking other forms of formal help, such as traditional medicine and fortune tellers, were advised by mothers to care for the depression itself, and not its possible causes.) (Self-help: simply cope with the problems on one's own, and in some cases advice to not bother others with one's own troubles, and even conceal the problems from others)
11	Common mental disorders among women, social circumstances and toddler growth in rural Vietnam: a population-based prospective study	J. Fisher, T. Tran, T. T. Nguyen, H. Nguyen and T. D. Tran (2015) [31]	To examine the effect of maternal common mental disorders (CMD) and social adversity in the post-partum year on toddler’s length-for-age index in a rural low-income setting	Baseline: Women in late pregnancy or 4–6 weeks post-partum Followed up (15 months later): the women and their toddlers	A population-based prospective cohort study	211	6/116 communes in Ha Nam randomly selected	DSM. Psychiatrist-administered. Structured clinical interviews for Diagnostic and Statistical Manual of Mental Disorders, Fourth Edition Diagnoses	Baseline: Women in late pregnancy or 4–6 weeks post-partum Followed up (15 months later): the women and their toddlers		Stunting prevalence among children in the first years of life in low- and middle-income settings. (LAZ < − 2) was 15.6% Maternal CMDs at follow up are related to diminished early childhood growth (LAZ) (Regression coefficient = − 0.15, 95% CI − 0.28 to − 0.05) Maternal CMD at baseline were indirectly related to toddler LAZ via maternal CMD at follow-up (regression coefficient = − 0.05, 95% CI − 0.11 to − 0.01)
12	Cross-cultural experiences of maternal depression: associations and contributing factors for Vietnamese, Turkish and Filipino immigrant women in Victoria, Australia	Rhonda Small, Judith, Jane Yelland (2003) [32]	To investigate in an Australian study of immigrant women conducted 6–9 months following childbirth (a) the associations of a range of demographic, obstetric, health and social context variables with maternal depression, and (b) women's views of contributing factors in their experiences of depression	318 Vietnamese, Turkish and Filipino women	Quantitative	318	Victoria, Australia	EPDS, Mental Health Sub-Scale (Cardona et al. 1995)	6–9 month postnatal	Isolation (including being homesick)-29%; lack of support and marital issues-25%; physical ill-health and exhaustion-23%; family problems-19%, and baby-related issues–17%	
13	One foot wet and one foot dry: transition into motherhood among married adolescent women in rural Vietnam	Klingberg-Allvin M, Binh N, Johansson A, Berggren V. (2008) [33]	To explore married Vietnamese adolescents' perceptions and experiences related to transition into motherhood and their encounter with health care service	Women younger than 20	Qualitative	22 women, 10 were carried out with pregnant women and 12 with newly delivered mothers	Study participants were recruited from 3 of the 25 communes in a rural district. Women were selected from lists provided by the staff at the CHC in each commune	Open-ended qualitative interviews covering 3 main areas: (a) adolescent women’s experiences in relation to the process of childbearing and transition to motherhood, (b) their attitude toward spacing and usage of contraception, and (c) their perceptions of the encounter with health care providers	Either pregnant or postnatal	(1) Ambivalence (both feelings of happiness and pride to be able to conceive and feelings of worry and fear of complications and a lack of confidence to cope with the processes of pregnancy and motherhood) in becoming a young mother; (2) Being in the hands of others; (3) Being ignored and patronized by the health care providers	
14	Postnatal depression and social supports in Vietnamese, Arabic and Anglo-Celtic mothers	Stuchbery, M., Matthey, S. & Barnett, B. (1998) [34]	To examine which deficits in components of women’s social support network are associated with postnatal depression	Anglo-Celtic, Vietnamese, and Arabic women	Qualitative	126 and 113 respectively for Vietnamese, 125 and 98 for Arabic and 128 and 105 for Anglo-Celtic mothers	Antenatal clinics at four public hospitals in southwestern Sydney, Australia	EPDS	6 weeks postpartum	Poor quality of the relationship with her partner and wanting more practical support from her partner were associated with higher scores on the EPDS	
15	Perceptions and experiences of perinatal mental disorders in rural, predominantly ethnic minority communities in northern Vietnam	Daniel Abrams, Liem T. Nguyen, Jill Murphy, Younji (Angie) Lee, Nhu K. Tran & David Wiljer (2016) [35]	To investigate knowledge/experiences and perceptions of perinatal mental disorders (PMDs) and their treatments at the community level in a rural, predominantly ethnic minority region of northern Vietnam	Primary health workers (PHWs) working at local community health centers, and pregnant or postpartum women enrolled in a program for maternal and infant health	Qualitative semi-structured interviews	14 perinatal women and 12 PHWs	Four communities located within the Dinh Hoa district of Thai Nguyen province	Two vignette scenarios, one based on DSM-IV	Either pregnant or women in their first-year postpartum	- Wandering around outside’, ‘attacking someone’, and ‘speaking without meaning’ - Changes in sleep and appetite - Mothers, however, were more likely than PHWs to refer to thoughts or emotions when speaking about mental health. The most commonly mentioned symptoms in this category related to worry, anxiety, or stress	PHWs’ experience with mental health patients of any kind was reported to be mostly limited to the long-term management of patients with psychosis or epilepsy, and very few reported diagnosing a new mental health patient within the past year
16	The clinical symptoms of postpartum depression	Dinh VH, Pham NT. (2022 [36]	To study the clinical features of postpartum depression	Postpartum depression patients treated at the Department of Psychiatry	A cross-sectional descriptive retrospective	31	Department of Psychiatry, 103 Military Hospital	Beck Depression Inventory		Symptoms of decreased mood, loss of interest and enjoyment, insomnia accounted for 100%. Fatigue, pessimistic patients accounted for 93.55%. 29.04% of patients have delusions of self-incrimination, 80.65% of patients having suicidal ideation	
17	Prevalence of postpartum depression and related factors at National Hospital of Obstetrics and Gynaecology	Ky, N.V.; Bac N.Q. (2021) [37]	To investigate prevalence, symptoms and risk factors of postpartum depression among women delivering at National Hospital of Obstetrics and Gynaecology	women delivering at National Hospital of Obstetrics and Gynaecology	A cross-sectional descriptive retrospective		National Hospital of Obstetrics and Gynaecology			feeling very bored/depressed, sad (83.3%), no longer interested in meeting or meeting with anyone (40.5%), feeling tired all the time (76.2%). Common symptoms of TCSS are: decreased attention span (71.4%), decreased self-esteem and confidence (52.4%), idea of guilt, unworthiness (80.9%), bleak future outlook, pessimism (73.8%), sleep disorder (100%), eating disorder (97.6%), in particular, there was one case of suicidal ideation and behavior (2, 38%)	

## Results

Overall, after titles were screened, abstracts were reviewed, and full texts were accessed, a total of 39 articles were included. Of these, 21 articles pointing out symptoms/experiences, consequences, and management of PPD among Vietnamese participants were kept for critical appraisal using JBI’s checklists. Four articles that did not meet quality for review after critical appraisal were excluded after reviewing; thus, 17 publications remained for further data analysis.

### Study characteristics

Research findings regarding symptoms, consequences and/or management of PPD of mothers in the postnatal period were reported intermittently during previous years in Vietnam, as shown in Table 2. Research reports (*n* = 5) that examined mothers’ symptoms and feelings, as well as PPD management used the qualitative method, while quantitative studies (*n* = 11) described the percentage of each symptom in PPD. Among studies reviewed, most focused on a 1–3-month period while only two mentioned mothers’ experiences during the first 24–48 h postpartum. Research settings varied in level of size from commune, district, province, to country. Several different research methods were used to examine PPD, including semi-structured, survey and other self-report scales.

**Table 2:** Characteristics of articles on PPD symptoms and management in Vietnam

Characteristics	Frequency (*n* = 17)	Percentage (%)
**Year**		
Before 2015	8	47.1
2016–2022	9	52.9
**Setting level**		
Commune	4	23.5
District	4	23.5
Province/City	3	17.6
Country	6	35.3
**Design**		
Quantitative	11	64.7
Qualitative	5	29.4
Mixed method	1	5.9
**Time of measurement (postnatal)**		
24 h-48 h	2	11,8
1–3 months	7	41,2
> 6 months	8	47,1
**Data source**		
Survey	11	64.7
Interviews	6	35.3

### PPD symptoms among Vietnamese participants

Symptoms of PPD often include a combination of: mood changes like anxiety, irritability, and a feeling of being overwhelmed; physical disorders such as sleep disturbance (beyond that associated with the care of the baby) or loss of appetite; self-criticizing or obsessive preoccupation with the baby’s health and feeding [9, 38]. This scoping review grouped symptoms reported in the included studies into three commonly found categories: *(1) physical symptoms; (2) emotional symptoms; and (3) abnormal behavior changes*, as shown in Table 3*.* Among physical symptoms, poor appetite was reported at a high percent [9, 35, 39]. Heart palpitations/Breathing difficulty/Nausea/Headaches/Constipation were also mentioned in one qualitative study as possible symptoms [24].

**Table 3:** PPD experiences and symptoms among Vietnamese participants

Theme	Sub-theme	Frequency mentioned in qualitative research	Percentage reviewed in quantitative research
**Physical symptom**	Poor appetite	2	75.5%; 97.6%
	Sleep disturbance	3	23%; 100%
	Heart palpitations/ Breathing difficulty/Nausea/Headaches/Constipation	1	< 10%
**Emotional symptom**	Nervous	2	9%; 20%; > 75%
	Sadness		13.3%; 43.3%; 83.3
	Unhappy/Tired	1	> 75%; 93.55%; 76.2%
	Thoughts of suicide/abusing child		37.9%;80.65%; 80.9%
	Feeling isolated: Lack of care from others Feeling useless	4	10%; 29%; 25%; 40.5%
**Abnormal behaviors**	Wandering around outside/ Attacking someone/ Speaking without meaning	1	
	Nighttime waking apart from infant care	1	25–58%

In terms of emotional symptoms reported with a wide range of prevalence in quantitative research, those most frequently mentioned were worries, anxiety, and stress. “*Feeling nervous*” was pointed out in three studies at various rates [7, 9, 27], while “*sadness*” was also a prominently featured concerned feeling [27, 36, 39]. Moreover, feelings of isolation included “*I am abusing my child*”, “*do not have time to interact with the child in a relaxed mood*” [7, 39], the thought “*they are useless and do not want to become a burden for others*” [30] or even “*being homesick*” [32]. From another perspective, mothers who lack care from others may have feelings of “*being ignored; being denied support, and being exposed to controlling behaviors*” [26, 39] or of “*living under pressure with the psychic force of household tensions or the weight of kinship conflicts*” [25]. A significant number of mothers had more severe symptoms in which 37.9% of them “*had suicidal thoughts*” [8, 36, 39].

Abnormal behaviors like “*wandering around outside*”, “*attacking someone*”, and “*speaking without meaning*” were not common but may be observed in some mothers who experienced a moderate level of PPD [35]. Nighttime waking apart from infant care was self-reported among women at high risk of PPD [24].

### Consequences and management of PPD

The consequences of PPD have an impact on both the mother and her child(ren). Mothers could face a higher risk of future common mental disorders [29], or in some severe cases, mothers suffering from PPD may have suicidal thoughts [8]. As summarized in Table 4, only two studies mentioned child stunting (the lowest extreme of child growth defined as height-for-age below − 2 standard deviations from the median of the reference population) due to the mother’s PPD which could be explained by interfering with sensitive-responsive parenting practices [28, 31].

**Table 4:** Management and consequences of PPD among Vietnamese mothers

**Consequences of PPD**	- Children stunting in the first year [28, 31]
	- Diminished early childhood growth [31]
	- Risk of long-term mental disorders among women with PPD [29]
	- Suicidal thoughts among PPD women in the previous seven days [8]
**Management of PPD**	- Psychiatric treatment and care were seldom recommended by health care workers. Mothers sought help from traditional medicine and fortune tellers [30]
	- More than two-thirds of women with common mental disorders at baseline had recovered by the follow-up assessment. None received formal mental healthcare [29]
	- Mostly limited to long-term management of patients with psychosis or epilepsy [35]

Three of the 17 articles discussed the management and follow-up of PPD in Vietnam [29, 30, 35]. Non-psychotic PPD required proper care including timing screening, early interventions, and follow-up assessment in order not to worsen to the more severe situations. However, the results of these three studies indicated insufficient mental healthcare services for mothers during this sensitive period when the postpartum required the most attention to be cared for. For women with severe depressive symptoms such as psychosis or epilepsy, long-term management was limited in Vietnam. As such, mothers having to find a strategy to help themselves tended to “*seek help from traditional medicine and fortune-tellers*” rather than to explore what advice was available from healthcare providers [30]. Follow-up after delivery and long-term management of PPD has been a significant maternal healthcare gap in Vietnam over the past decade.

## Discussion

Seventeen articles examining symptoms of mothers and management of PPD in postnatal period studies with different timeframes were identified and included in this scoping review of depressive symptoms, consequences, and management of PPD among mothers in Vietnam. Here, publicly accessible information on the experiences and management of women suffering from PPD is not regularly updated, in contrast to the practice in some other countries [16, 40]. This important maternal health issue urges a call for future studies on PPD among Vietnamese women with a primary focus on early detection, and its barriers to and enablers of commonly reported symptoms, rather than on the prevalence of PPD.

One of the few published studies reported that women experienced high levels of depressive symptoms in the two-week postpartum period, peaking on day five [41]. During these early days following delivery, women experienced mood disturbances, including emotional lability, frequent crying, anxiety, fatigue, insomnia, anger, sadness, and irritability [41]. In this scoping review, two studies investigated symptoms 24–48 h after birth, but little was revealed about this initial period, especially after day 2 in the first week. Examination of early PPD symptoms during these very first days is needed to support interventions for high-risk women during these days of the most sensitive moods disorders [42, 43].

Herein, our review delineates the results of studies on PPD symptoms into those that are physical, those that are emotional, and abnormal behaviors. A group of emotional symptoms was identified in an earlier review [35]. Previous studies on women in other countries confirmed that this emotional phenomenon is accompanied by symptoms such as mood disturbances ranging from euphoria to sadness, high sensitivity, sudden crying, restlessness, poor concentration, and anger [44]. Additionally, a systematic review of qualitative evidence regarding new mothers’ experiences of PPD revealed four themes: inability to control feelings; ambivalence towards family members; imbalanced support between demands; and expression of hopelessness/helplessness [16]. Research from around the world found that hormonal changes after delivery, the stress to the mother of caring for her baby, or even situations surrounding labor may each lead women to experience physical symptoms, such as eating disturbances or sleeping disorders [41, 45]. One study examining the experiences of maternal and child health nurses responding to women with PPD in Australia showed agreement regarding symptoms such as lack of feeling and lack of concentration [46]. Similarly, according to DSM-IV diagnostic criteria, insomnia, changes in appetite, and suicidality were assessed as indicative of a major depressive disorder [2]. While not frequently reported in Vietnam, some abnormal behaviors like “*wandering around outside*”, “*attacking someone*”, “*speaking without meaning*”, and “*nighttime waking*” were observed in previous studies elsewhere, especially among patients with a severe mental disorder [47, 48]. Despite the categorization of PPD symptoms into three groups of physical, emotional, and behavioral symptoms, this scoping review indicates that previous studies among the Vietnamese population did not pay sufficient attention to early recognized symptoms that started after birth. This paucity of information, along with psychosocial barriers, causes difficulty for both clinical practitioners and family members to assess early and thereby prevent the occurrence of PPD symptoms.

Appropriate management and follow-up are crucial to reducing the harmful effects of PPD and may positively impact treatment outcomes. Different types of mood disorders after giving birth required various follow-up treatment. While postpartum blues is believed to disappear without treatment, the overlooked situations can result in non-psychotic PPD, which may need professional care, and even the most severe situation as postpartum psychosis will require hospitalization [3, 38, 44]. The differentiation between PPD and postpartum blues was discussed in only three studies included in our review [29, 30, 35]. The lack of concentration on PPD cases, especially severe ones, can lead to inadequate care and further treatment for these women. Although postpartum management and care for mothers and infants were not examined well in Vietnam, these issues were explored in other countries [12, 13]. Women receiving interventional support and monitoring demonstrated better maternal mental health outcomes compared to the control group. The importance of support to mothers was examined in a separate study [13], which confirmed that primary care-based screening, diagnosis, and management improved mothers’ depression outcomes at 12 months. Postpartum Vietnamese women are in urgent need of timing and comprehensive maternal healthcare services; however, to provide such support, intervention programs need to be well-informed by local research that follows mothers along a postnatal timeline.

In this scoping review of articles published on PPD in Vietnam, both mothers’ and child(ren)’s health problems are mentioned, including suicidal thoughts and further mental disorders in mothers, and stunting and slow growth in children [28, 31]. PPD was recognized as a systemic issue affecting a woman’s functioning, sense of well-being, family relationships, parenting capacity, and competency to control her daily life [41]. Further impacts should be investigated to capture more completely the consequences of PPD on Vietnamese mothers. This would provide evidence for informed decision-making on the implementation of suitable screening and management programs for this at-risk population. These programs should consider educating women and their family members to follow modern, evidence-based methods to manage PPD rather than relying on ‘fortune-teller’ advice or ‘word-of-mouth’ practices.

Several key questions regarding PPD in Vietnam remain and offer opportunities for future research. In particular, these include considering the underlying background of barriers to and enablers of early detection of symptoms of PPD and its follow-up management after screening or diagnosis. Mothers’ symptoms were reported without reference to a group of common combined symptoms or sequence of symptoms mostly in the context of a private medical examination, with limited public awareness through community engagement [21]. Community mental health stigma was studied as a significant barrier to access follow-up healthcare services [49], and was associated with lower acceptance of treatment [50]. In addition to the unique psychosocial aspects within the Vietnam context, this is challenging for healthcare workers and family members to detect early signs or symptoms of PPD and to provide in-time support for these women. Furthermore, no research on typical consequences affecting Vietnamese mothers was found. This suggests a pressing need for a follow-up study to identify possible consequences of PPD and support strategies required both for postpartum mothers in Vietnam and to increase community awareness. Similarly, a management program or follow-up plan after early detection is recommended to focus on mothers both at mild stages and during more severe periods of PPD.

## Strengths and limitations

This scoping review provides a perspective of PPD symptoms, consequences, and management in Vietnam. Published articles were searched on qualified databases, filtered, and critically appraised using a validated process provided by JBI. However, little evidence has been collected in Vietnam, especially in recent years. Additionally, many unpublished studies have not been posted online or otherwise widely shared. These resources were therefore not included in the review due to their limited accessibility. This issue may narrow the scope of our current understanding of the research topic. Finally, article selection was by reading and reviewing their content using the JBI checklist, but which did not follow fully the JBI systematic review process. This led to difficulty comparing and synthesizing research findings due to a shortage of local Vietnamese databases.

## Conclusion

This scoping review presents an overview of research exploring PPD in Vietnam. Highlighted symptoms mentioned are sadness, tiredness, being ignored, lack of interest in the baby, loss of appetite, and sleep disturbance. The PPD symptoms described, categorized as either physical symptoms, emotional symptoms, or abnormal behavior changes, are recommended to facilitate further research on early screening for PPD among postpartum women. This study highlights the limited information that is currently available on the consequences of PPD among Vietnamese mothers and the management of PPD in Vietnam. We therefore advocate for in-depth studies on these topics to be conducted.

Early detection of PPD and long-term follow-up play essential roles in effective treatment of this common mood disorder. A focus should be on recognizing symptoms during early postnatal days to enable community education programs to be rolled out successfully. Identification of PPD consequences on mothers is strongly recommended, as is a strategy for hospitals and healthcare services to follow up with women reporting negative feelings or sleep disturbance. These local community-based initiatives should also be culturally aware, such as acknowledging the ritual belief system of Vietnamese mothers seeking to resolve depressive feelings after giving birth.

## Data Availability

The datasets used and analyzed during the current study are available from the list of articles in Table 1.
